# Double trouble: cytosolic and nuclear IKKα in cancer

**DOI:** 10.1098/rsob.240375

**Published:** 2025-08-06

**Authors:** Kirsty Tinto, Margaret Cunningham, Robin Plevin

**Affiliations:** ^1^ Strathclyde Institute of Pharmacy and Biomedical Sciences, University of Strathclyde, Glasgow, UK

**Keywords:** cytosolic, nuclear, IKKα, cancer, NF-κB

## Abstract

IκB kinase alpha (IKKα) is a serine/threonine kinase originally known for its role in nuclear factor kappa-light-chain-enhancer of activated B cells (NF-κB) signalling, which integrates inflammatory processes and cancer. IKKα can function within the IKK complex in canonical NF-κB signalling, alongside its homologous family member, IKKβ, and regulatory subunit IKKγ (NEMO). However, a key role for IKKα is its ability to promote non-canonical NF-κB signalling. Additionally, the dynamic ability of IKKα to shuttle between the cytosol and nucleus, mediates NF-κB-independent effects which further its role in inflammation and tumour progression. More recently, an endosomal-generated, nuclear-active IKKα isoform, p45-IKKα has been discovered and implicated in cancer and chemoresistance. This review focuses on current knowledge of the complex and intricate roles of nuclear and cytosolic IKKα in promoting tumour progression. By highlighting the molecular roles of IKKα in several cancer subtypes, and its integral roles in many of the hallmarks of cancer throughout this review, we highlight the therapeutic potential of IKKα as a future anti-cancer drug target.

**Figure:**
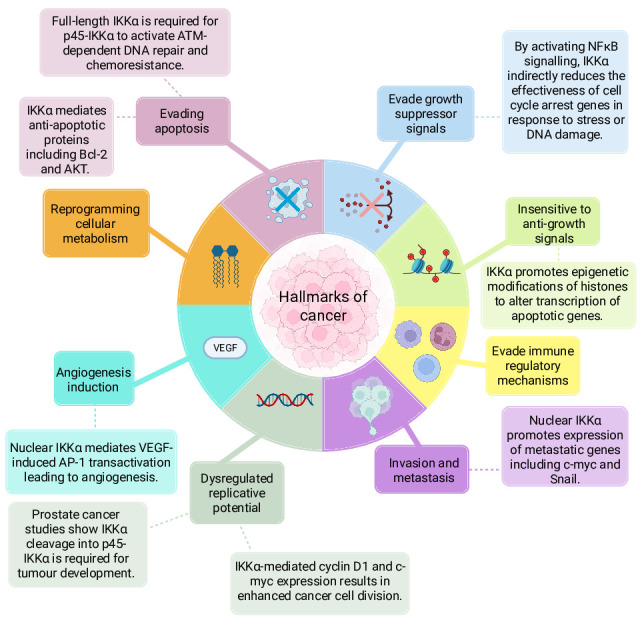

## 1. General introduction

Despite extensive efforts to conduct research into cancer aetiology, including the production of expansive data and signalling networks of genes and proteins [1], cancer remains the leading cause of death in 57 countries [2]. Additionally, cancer has the highest economic burden globally out of the 15 highest causes of mortality [3]. There are currently eight characterized hallmarks of cancer, which include proliferative signalling, evading growth suppressors, evading cell death, enabling replicative immortality, inducing angiogenesis, activating invasion and metastasis, reprogramming energy metabolism, and avoiding immune destruction, each contributing to complex cancer biology [4]. To overcome the detrimental effect which cancer places on the economy and health, more research and treatment options are required. Given the associated adverse effects associated with current radiotherapy and chemotherapy approaches, a targeted molecular pharmacological approach would be beneficial to potentially negate the off-target and associated unfavourable treatment-related toxicity [5,6].

The ever-expanding knowledge of proteins involved in oncogenesis is making such precision therapies increasingly feasible. IκB kinase alpha (IKKα), a member of the IκB kinase family, is vital for cell differentiation and survival, and therefore integral in orchestration of physiological homeostasis [7]. However, IKKα also contributes to the molecular mechanisms which induce cancer development and systemic metastasis [8]. The central role of cytosolic IKKα (85 kDa) is in mediating non-canonical nuclear factor kappa-light-chain-enhancer of activated B cells (NF-κB) signalling [9]. Recently, the truncated nuclear active IKKα isoform, p45-IKKα has also been shown to promote oncogene expression and chemoresistance. Few research groups have investigated the role of p45-IKKα in a cancer setting. However, the understanding of p45-IKKα function is still emerging. Nevertheless, targeting IKKα presents a potentially promising therapeutic strategy in cancer treatment.

## 2. Purpose of review

The purpose of this review is to provide an overview of the current understanding of full-length cytosolic and nuclear IKKα in several types of cancer. It outlines the complex and intricate molecular mechanisms which IKKα can play a role in the promotion of cancer hallmarks in colorectal, lung, prostate and skin cancers. In addition, it explores the emerging, though less extensively studied, cancer-related roles of the p45-IKKα isoform. Additionally, this review examines the current strategies which have been utilized to inhibit IKKα activity in cancer, highlighting the potential directions for future research.

## 3. NF-κB signalling pathways

NF-κB was initially identified in 1986 as a transcription factor by Ranjan Sen in David Baltimore’s laboratory using mobility shift assays to confirm its interaction with an 11 base pair sequence (GGGGACTTTCC) within the immunoglobulin κ light-chain enhancer of human B cells [10]. Since the initial discovery of NF-κB almost four decades ago, it has become clear that NF-κB is not exclusive to B cells. The NF-κB family is comprised five family members: p50 and p52 (processed from precursors p105 and p100), p65 (RelA), c-Rel and RelB. NF-κB signalling is required for many important physiological processes, including immune response, inflammation, metabolism and memory. These functions have been covered elsewhere [11,12], and therefore will not be covered within this review. However, despite being this crucial within immunological processes, NF-κB also enhances pathophysiological responses, by promoting the expression of pro-inflammatory and tumour-promoting genes, which subsequently promotes inflammatory-based diseases and cancer [13].

The IKK family plays an integral role in NF-κB signalling, which operates through two main pathways: canonical and non-canonical cascades. Canonical NF-κB signalling is activated by a plethora of pro-inflammatory stimuli, including pathogen-associated molecular patterns, damage-associated molecular patterns and pro-inflammatory cytokines, such as TNF-α and IL-1β [13–16]. Specific binding of these ligands such as IL-1β to their relevant receptors (IL-1R1) induces rapid activation of intracellular signalling, which triggers recruitment of adaptor proteins to the receptor’s cytoplasmic domain. This subsequently results in activation of a MAP kinase kinase kinase (MAP3K), Transforming growth factor-β-activated kinase 1 (TAK1) within 1−2 min and peak activation within 3−5 min after stimulation [17]. TAK1 can phosphorylate IKKβ or IKKα in the trimeric IKK complex, which is composed of IKKα, its closely related family member IKKβ and the regulatory subunit IKKγ [18–21]. However, the role of IKKα within the canonical NF-κB signalling pathway is limited. Therefore, most studies focus on the TAK1-mediated phosphorylation of IKKβ at Ser177 and Ser181, as this results in the phosphorylation and proteasomal degradation of Inhibitor of kappa B alpha (IκBα). This induces subsequent nuclear translocation of NF-κB p65 dimers with other NF-κB transcription factor members, most commonly being p50 NF-κB subunits [22].

In contrast, the non-canonical NF-κB signalling axis is dependent on IKKα homodimers and is IKKβ and IKKγ independent [23]. This pathway is activated more slowly by a group of specific TNF superfamily members, including lymphotoxin, CD40L and tumour necrosis factor superfamily member 14, also known as LIGHT. By binding to their respective receptors, these ligands induce recruitment of adaptor protein TRAF3 which is bound to TRAF2 and cIAP1/2, to the receptor’s cytoplasmic domain. In a basal state, cIAP1/2 ubiquitinates NF-κB-inducing kinase (NIK, also known as MAP3K14) at Lys48 within its TRAF3 binding domain which targets NIK to the proteasome, resulting in its degradation. However, when the non-canonical NF-κB cascade is activated by ligand-receptor binding, this results in upregulated intracellular NIK levels, enabling liberated NIK to phosphorylate IKKα at Ser176 and Ser180 within its activation loop. The dependence on protein synthesis (NIK accumulation) for non-canonical signalling results in a notably slower signalling pathway than observed in the canonical NF-κB axis [24]. In collaboration with NIK, phosphorylated IKKα sequentially phosphorylates a large IκB p100 at conserved serine residues Ser866 and Ser870. In turn, this results in recruitment of the SCFβ-^trcp^ ubiquitin ligase complex, which subsequently induces ubiquitination at Lys856, the ubiquitin acceptor site on p100, leading to p100 degradation at the proteasome leading to p52 production. As a result, p52 can form an active NF-κB heterodimer with RelB, which undergoes nuclear translocation to promote specific anti-apoptotic and pro-inflammatory genes.

**Figure 1.**
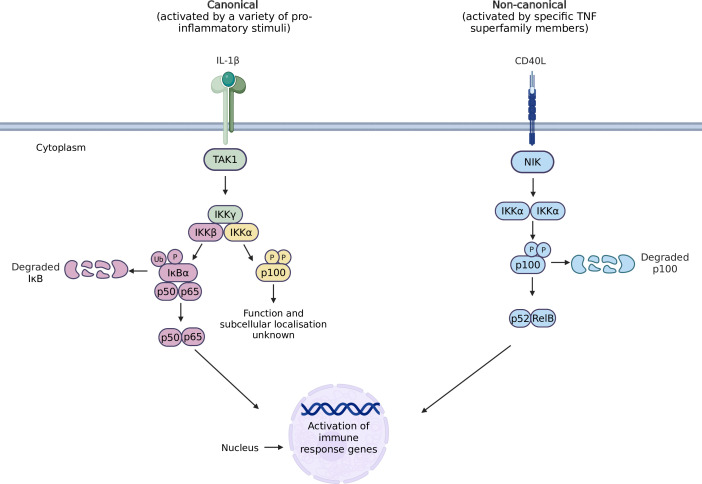
A diagrammatical representation of the canonical and non-canonical NF-κB signalling pathways. Canonical NF-κB signalling is stimulated by a plethora of inflammatory cues, while non-canonical NF-κB signalling is activated by specific TNF-superfamily members. Canonical NF-κB signalling relies on activation of the IKK complex, consisting of IKKα, IKKβ and IKKγ. IKKβ phosphorylation induces phosphorylation and degradation of IκBα, and downstream nuclear translocation of p50-p65 NF-κB heterodimers. IKKα phosphorylation results in rapid phosphorylation of p100, another NF-κB family member, but the subsequent downstream events remain unexplored. Non-canonical NF-κB signalling is dependent on IKKα homodimers, leading to phosphorylation of p100, resulting in its proteasomal degradation into p52. This facilitates the formation of p52-RelB heterodimers, which translocate to the nucleus. Both signalling pathways promote distinct subsets of pro-inflammatory and oncogenic genes. Original figure created on BioRender.com.

The focus of canonical and non-canonical NF-κB signalling, and the model of this pathway appears to be changing, as emerging evidence focuses on IKKα as a viable drug target for oncogenesis. Recent studies have indicated a role for IKKα in IL-1β-mediated canonical NF-κB signalling leading to rapid non-canonical NF-κB2 (p100) phosphorylation [25]. Intriguingly, this phosphorylation event occurs much earlier after stimulation than the activation typically observed during non-canonical NF-κB signalling induced by TNF-superfamily members [25,26]. This newly discovered IKKα-mediated arm of canonical NF-κB signalling is TAK1-dependent, NF-κB-inducing kinase (NIK)-independent and p100 phosphorylation does not appear to result in p100 proteasomal degradation or the generation of p52 [25,27]. Therefore, the role of IL-1β-induced IKKα-dependent p100 phosphorylation is likely to result in functions independent of the non-canonical p52-RelB heterodimers. Further research into this signalling pathway may provide further insight into the diverse roles of IKKα in cancer. A summary of canonical and non-canonical NF-κB signalling is highlighted in figure 1.

## 4. The activation of p45-IKKα in cancer

A surprising discovery was that IKKα can exist as a truncated isoform, p45-IKKα, which has since become an emerging focus due to its involvement in cancer biology. Upon DNA damage, the v-Raf murine sarcoma viral oncogene homolog B (BRAF)-TAK1-p38/MAPK axis phosphorylates p45-IKKα, which is generated in endosomes, facilitating its nuclear translocation [8,28,29]. Since the discovery of this proteolytic nuclear p45-IKKα, its functions have been investigated in several colorectal cancer (CRC) cell-based studies [8,28,30,31]. However, p45-IKKα remains a likely underreported isoform in cancers overall. In recent years, nuclear IKKα has attracted attention due to its dynamic role in regulation of DNA damage response genes, and its ability to drive chemoresistance in cancer [29]. Still, much remains to be understood about the broader functions and relevance of p45-IKKα across diverse cancers.

## 5. The structure of IKK family members

Structurally, IKKα is an 85 kDa kinase with a modular structure that enables its diverse functions in NF-κB signalling. The N-terminal kinase domain drives its catalytic activity, while the scaffold/dimerization domain, which contains a leucine zipper, facilitates the formation of IKKα homodimers and heterodimers with IKKβ. This is essential for complex assembly and downstream signalling. IKKα also possesses a helix–loop–helix motif, and a C-terminal NEMO-binding domain which enables IKKα–IKKγ binding as part of the IKK trimeric complex in canonical NF-κB signalling [32,33].

IKKβ is an 87 kDa kinase which features many structural similarities to IKKα, including a kinase domain with two phosphorylation sites required for its activation (Ser177 and Ser181), a scaffold/dimerization domain and a NEMO-binding domain. In addition to these shared elements, IKKβ contains a unique ubiquitin-like domain. This additional domain is vital for its involvement in IκBα-driven canonical NF-κB signalling [34].

**Figure 2.**
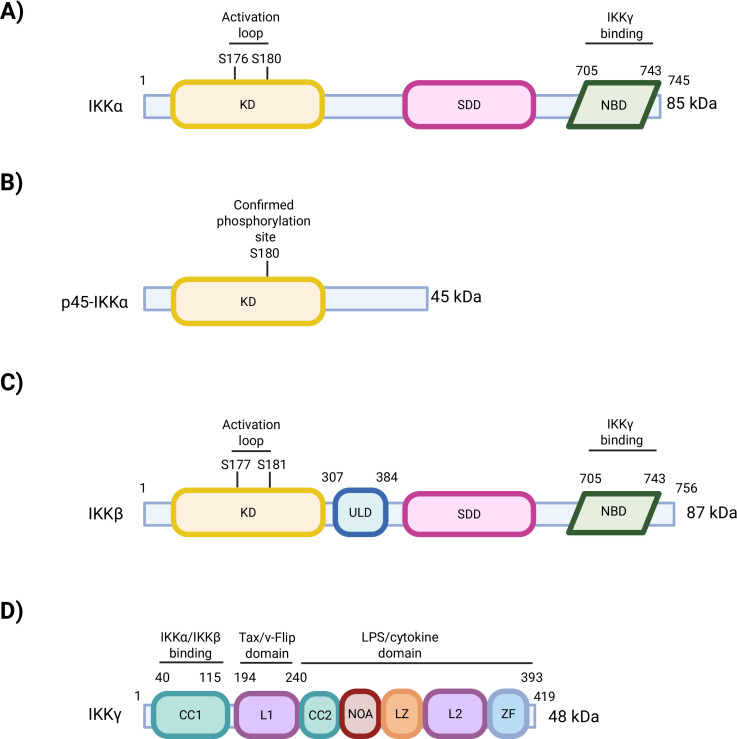
Structural representation of the IKK family member subunits. (A) The structure of IKKα comprised kinase domain with two phosphorylation sites, Ser176 and Ser180, as well as a scaffold dimerization domain and a C-terminal NEMO-binding domain, which facilitates IKKα–IKKγ binding. (B) The structure of an endosomal-derived, nuclear active IKKα isoform, p45-IKKα. This truncated form has been shown to be phosphorylated at one site by TAK1, Ser180 and lacks the other regulatory domains which IKKα possesses. (C) IKKβ possesses a closely related structure to IKKα, containing a kinase domain with two phosphorylation residues, Ser177 and Ser181, responsible for its action, a scaffold dimerization domain and a NEMO-binding domain. IKKβ also contains an additional ubiquitin-like domain. (D) IKKγ contains a coiled coil 1 domain involved in IKKα/IKKβ activation when bound, linker 1 which is a Tax/v-FLIP domain and LPS/cytokine domain consisting of coiled coil 2, ubiquitin binding domain, leucine zipper, linker 2 and another ubiquitin binding domain known as zinc finger. KD, kinase domain; ULD, ubiquitin-like domain; SDD, scaffold dimerization domain; NBD, NEMO-binding domain; CC1, coiled coil 1; L1, linker 1; CC2, coiled coil 2; NOA, ubiquitin binding domain; LZ, leucine zipper; L2, linker 2; ZF, zinc finger. Original figure created on BioRender.com.

Notably, unlike IKKβ which is predominantly cytoplasmic, IKKα has a nuclear localization signal (amino acid residues 232−240) within its kinase domain, enabling IKKα to traverse between the cytoplasm and nucleus [35]. IKKα undergoes nuclear translocation and acts as an acetylase and chromatin kinase to modify histones and regulate chromatin structure. In turn, this results in transcription of inflammatory genes and DNA repair [5,36–38]. In contrast, p45-IKKα, which is formed by proteolytic cleavage of IKKα, contains the kinase domain, but lacks regulatory domains, including the leucine zipper, helix−loop−helix motif and NEMO-binding domain observed in cytosolic IKKα structure [30]. IKKγ is a 48 kDa kinase composed of several domains, including the coiled coil 1 domain which facilitates oligomerization by enabling IKKγ-IKKα and IKKγ-IKKβ binding. A diagrammatical representation of the structure of the classical IKK family members (IKKα, IKKβ and IKKγ), and p45-IKKα are illustrated in figure 2.

## 6. IKKα in colorectal cancer

CRC is the fourth most frequently diagnosed cancer, accounting for 6.1% of cases, following lung (11.6%), female breast (11.6%) and prostate cancer (7.1%) [39]. It is also the second leading cause of cancer-related mortality, responsible for 9.2% of deaths, second only to lung cancer, which accounts for 18.4% [39]. Due to this significant health burden, increasing attention has been directed towards understanding both NF-κB-dependent and NF-κB-independent functions of IKKα in CRC. Studies using CRC cell lines have contributed to a more detailed understanding of IKKα’s role in this malignancy. TNFα- and IL-1α-induced p65 phosphorylation (S468) and subsequent nuclear translocation is inhibited in IKKα knockout clones of an epithelial colorectal cell line (HCT116 cells) [40]. Interestingly, IKKβ knockout HCT116 clones had no effect on p65 phosphorylation in this study. This intriguing finding highlights a dominant role for IKKα over IKKβ in CRC in cell-based studies, which is somewhat unusual, as p65 phosphorylation is traditionally believed to be more dependent on IKKβ [40]. This suggests that the roles of the IKKs in canonical signalling are cell- and region-specific and justifies the need for additional research into the role and therapeutic potential of IKKα in CRC.

IKKα is highly expressed in colorectal tumours and is recruited to the promoter regions of several Notch-dependent genes, including *hes1, hes5* and *herp2/hrt1*, resulting in their transcriptional activation. IKKα phosphorylates the gene corepressor Silencing Mediator of Retinoic acid and Thyroid hormone receptor (SMRT) at Ser2410, resulting in dissociation of SMRT from Notch target genes, and subsequent abnormal SMRT displacement from the nucleus to the cytoplasm. This enables Notch target gene-mediated cancer cell survival, proliferation and chemoresistance [41,42]. IKKα inhibition by a non-selective IKKα and IKKβ inhibitor, BAY 11-7082, and dominant-negative IKKα expression enables the reassociation of SMRT with Notch-dependent genes, resulting in suppression of these target genes [43,44]. Additionally, BAY 11-7082 treatment decreases tumour size in CRC xenografts implanted in nude mice which demonstrates the therapeutic potential for targeting IKKα and IKKβ in CRC [44].

P45-IKKα has primarily been studied in CRC cell lines, likely due to its high abundance in CRC cells carrying the BRAF Val600Glu (V600E) mutation, which results from a T1799A transversion in exon 15 of the BRAF gene (previously referred to as T1796A) and causes a substitution of valine with glutamic acid at codon 600 (formerly Val599Glu) [28,45,46]. This mutation causes constitutive activation of the BRAF kinase domain, which leads to dysregulated downstream signalling [47]. However, it is worth noting that p45-IKKα is not exclusively activated in these conditions and can also be induced in wild-type CRC cells following activation of the BRAF-TAK1-p38-MAPK axis [29].

Once activated, p45-IKKα promotes ATM phosphorylation and activation, and initiates subsequent DNA repair processes [29]. This function contributes to reduced sensitivity to both chemotherapy and radiotherapy in CRC cells [29]. Notably, cleavage of full-length IKKα to p45-IKKα is crucial to induce cancer cell survival and growth in human CRC cell lines and in mouse models [30]. Importantly, the role of p45-IKKα appears to be NF-κB independent [28,31]. This has been demonstrated through the use of endosomal acidification inhibitors (chloroquine or bafilomycin A1), which diminish p45-IKKα phosphorylation without affecting NF-κB function [28,31]. However, despite its activation being independent of the NF-κB pathway, p45-IKKα chromatin-binding capabilities are dependent on the presence of full-length IKKα [30]. This is particularly interesting as both full-length IKKα and p45-IKKα are central players in DNA damage responses. Full-length IKKα induces STAT3 activation via the cytokine, leukaemia inhibitory factor (LIF) by NF-κB-dependent mechanisms and further, the JAK/STAT pathway resulting in downregulation of early apoptosis, chemotherapeutic resistance and a poor prognostic outcome in CRC [48]. Interestingly, IKKα also directly phosphorylates BRD4 at Ser1117, enabling BRD4 chromatin binding to STAT3 target genes and activation of ATM, to promote DNA repair and subsequent therapy resistance [48]. Consistent with its role in chemoresistance, in SW-480 colon cancer cells treated with doxorubicin, IKKα siRNA knockdown increases cancer cell apoptosis by enhancing reactive oxygen species (ROS) levels and ROS-mediated damage within the cells [49]. Following exposure to DNA damage-inducing agents, NEMO binds to a pre-bound IKKα-ATM complex [50]. Furthermore, NEMO is not required for ATM activation but plays a key role in the nuclear translocation of ATM and activated p45-IKKα, and their subsequent DNA repair mechanisms in CRC cells [50]. Consistently, bioinformatic analysis reveals a correlation between high expression of genes encoding NEMO, IKKα and ATM with poor patient prognosis in CRC [50]. Therefore, despite its NF-κB-independent activation, the role of p45-IKKα in DNA damage repair and tumour cell survival is dependent on both NEMO and IKKα.

## 7. IKKα in lung cancer

Non-small cell lung cancer (NSCLC) is responsible for 80–85% of all lung cancer diagnoses, with a 5 year survival rate of approximately 20% [51,52]. In lung adenocarcinoma, mutations in the V-Ki-ras2 Kirsten rat sarcoma viral oncogene homolog (KRAS) are observed over 33% of cases, and 45 600 new lung cancer diagnoses annually in the USA contain the four most common KRAS mutant alleles [53,54]. In a study depicting the role of IKKα and IKKβ in KRAS-mutant lung adenocarcinoma, IKKα enhanced both genetic and chemical KRAS-mutant lung adenocarcinoma, and high IKKα expression was observed in cells from urethane-treated *FVB* and Ad-*Cre*-treated *LSL.KRAS*^G12D^ (KRAS mutation) lung adenocarcinoma mice models [55]. In contrast, IKKβ expression was lower, and therefore IKKα appears to play a greater role in KRAS-mutant lung adenocarcinoma [55]. Another research group have shown that IKKα deletion in KRAS^G12D^ mice results in increased cancer cell growth, and decreased p53/p21 and cell senescence [56]. Deletion of IKKα was also found to upregulate NOX2 expression, resulting in increased ROS accumulation and heightened oxidative damage [56]. Under normal conditions, ROS levels are tightly regulated by antioxidant defence mechanisms, including those mediated by NRF2. In the context of IKKα deficiency, the NRF2-driven ROS scavenging system becomes even more critical for mitigating oxidative stress, particularly in KRAS^G12D^ mice with deletion of the IKKα gene compared to KRAS^G12D^ controls. Collectively, these findings suggest that IKKα plays a protective role by limiting excessive ROS accumulation and maintaining a balance that supports ROS-mediated antitumour activity. This dual function helps to suppress the initiation and progression of lung adenocarcinoma (ADC). The BRAF^V600E^ mutation, which was previously described above, is frequently observed in NSCLC, alongside CRC and melanoma, as documented in multiple review articles [57–59]. Despite this, the role of p45-IKKα remains uncharacterized in NSCLC to date.

In lung adenocarcinoma, IKKα promotes phosphorylation of dopamine- and cyclic AMP-regulated phosphoprotein (DARPP-32), leading to decreased inhibition of protein phosphatase 1, and subsequent ERK dephosphorylation. In turn, these events promote the ERK signalling pathway, and therefore enhance lung cancer development [51]. Additionally, in NSCLC, IKKα is thought to induce metastasis regardless of its subcellular localization [7]. Both cytosolic and nuclear IKKα enhance oncogenesis by activation of ERK, p38 and mTOR activation in this context. However, only cytosolic IKKα induces EGFR and NF-κB signalling in NSCLC cells. On the other hand, nuclear IKKα in its full structural conformation promotes c-myc activation, which is encoded by a gene which is overexpressed in over half of NSCLC cases [7,52]. Additionally, nuclear IKKα was observed to promote Snail activation in NSCLC cells, which has been linked to the development and metastasis of several cancers, including lung cancer, breast cancer and gastric cancer [7,52]. Furthermore, Akt can phosphorylate IKKα resulting in NF-κB activation, and subsequent increased production of Snail [60]. Cyclin D1, a key cell regulator predominantly located in the nucleus when inactive, is phosphorylated at residue T286 by IKKα leading to cyclin D1 dysregulation and wide subcellular distribution, which drives progression of lung and breast tumours [61–65]. Due to the more prevalent overexpression of cyclin D1 compared to other D-type cyclins in human cancers, development of selective IKKα inhibitors, also selective cyclin D1 inhibitors, may be beneficial for patients with specific types of lung cancer [66].

## 8. IKKα in skin cancer

The role of IKKα in skin cancer is complex, as some studies have indicated that IKKα negatively regulates cancer cell division in mice keratinocytes, and nuclear IKKα is required for skin development via NF-κB-independent mechanisms [8,67,68]. However, studies investigating the role of IKKα in skin cancer are limited compared to other malignancies, and findings are often contradictory. For instance, other studies have highlighted that full-length nuclear and cytoplasmic IKKα protein in mice keratinocytes are involved in oncogenesis in skin cancer [69].

Despite the contradictory evidence, IKKα appears to play crucial NF-κB-independent roles in skin cancer. Specifically, IKKα expression is enhanced by its binding to chromatin at the 14-3-3 theta locus which downregulates CDC25, a phosphatase which regulates G2/M stages of the cell cycle and is involved in the transfer of accurate DNA information to daughter cells [8]. In turn, insufficient CDC25 activity results in excessive cell proliferation and damaged or inaccurate DNA being transferred to daughter cells, which can enable the induction and progression of skin squamous cell carcinoma. Additionally, both nuclear and cytoplasmic IKKα enhance tumour cell migration and promote expression of MMP9, and anti-apoptotic proteins, Blc-2 and Akt. Additionally, IKKα downregulates apoptotic proteins in PDVC57 cells, which is a cutaneous squamous cell carcinomas (cSCC) cell line derived from C57BL/6 (B6) mice [70]. IKKα has also been demonstrated to be instrumental in ultraviolet B (UVB)-induced apoptotic responses in human keratinocyte (HaCat) and mouse embryonic fibroblasts (MEFs) in a p53/PERP-dependent and NF-κB-independent manner [71]. Additionally, full-length IKKα in the nucleus can bind c-Fos and phosphorylate histone H3 on the *vegf* promoter regions containing AP-1 to enable AP-1 transactivation, which promotes UVB-induced angiogenesis [72]. As UVB has a prominent role in UV-related DNA damage in skin due to its longer wavelength than UVA and UVC, IKKα may offer drug target validity in the future for skin cancer treatment [71].

## 9. IKKα in prostate cancer

According to data obtained in 2020, prostate cancer is the second most prevalent cancer-related death and the most frequently diagnosed cancer in the UK [73]. Early studies highlighted that silencing IKKα expression by two targeted siRNA sequences diminished the migratory ability of a prostate cancer cell line, PC-3 cells, by approximately 30% and 40% [74]. More recently, cytoplasmic and nuclear IKKα has been linked to reduced survival time expectancy following relapse in prostate cancer [75]. Additionally, roles for the RANKL-induced, IKKα-dependent non-canonical NF-κB signalling axis have been established in oncogene expression and chemically induced prostate and breast cancer development [76]. IKKα promotes the activity of mTORC1, which further enhances IKKα and IKKβ activity, and downstream NF-κB activity in PC-3 cells [77]. This suggests that IKKβ is a downstream kinase of IKKα in prostate cancer. Findings from other research groups have highlighted that accumulation of nuclear IKKα displays a key NF-κB-independent role in prostate cancer progression by suppression of anti-tumorigenic *Maspin* gene transcription, which would subsequently result in prostate cancer metastasis to the bone [65,78]. IKKα also phosphorylates S67 and S71 residues on c-myc, which enhances c-myc-induced anti-apoptotic effects in prostate cancer [79]. In concert, IKKα knockdown downregulates c-myc activity and increases phosphorylation of T58, which enables polyubiquitination of T58 and subsequent degradation of c-myc by GSK3β [79]. Similar to its role in CRC, IKKα but not IKKβ promotes nuclear export of SMRT in the prostate cancer cell line DU145 [80]. This is significant as p50:p50 NF-κB homodimers recruit SMRT to repress NF-κB target genes, such as *IL-8* and *c-IAP2*. IKKα-mediated phosphorylation of chromatin-bound SMRT leads to its functional inactivation and dissociation, reducing histone deacetylase 3 (HDAC3) activity at NF-κB-regulated promoters. This phosphorylation-driven derepression of SMRT enables recruitment of RelA/p65, p300 and RNA polymerase II, allowing p50:p65 NF-κB heterodimers to initiate transcription of target genes [80]. IL-8 plays a cancer-protective role in the prostate tumour microenvironment, as it facilitates infiltration of immunosuppressive polymorphonuclear myeloid-derived suppressor cells, which express the IL-8 receptor, CXCR2 [81]. This exemplifies the importance of IKKα in oncogene expression resulting in prostate tumour migration and metastasis.

The intricate roles of IKKα in prostate cancer are further expanded by the ability of IKKα to mediate crosstalk of signalling pathways. LcRNA-PCAT1 directly interacts with PH and leucine-rich repeat protein phosphatase (PHLPP), which removes PHLPP from a trimeric PHLPP/FK506-binding protein 51 (FKBP51)/IKKα complex. This results in downstream activation of Akt and NF-κB signalling pathways in castration-resistant prostate cancer, which merits research into PCAT1, but also IKKα as a therapeutic drug target for prostate cancer [82].

## 10. IKKα as a drug target

In early studies, highly selective ATP-competitive IKKβ inhibitors were developed to be tested therapeutically. However, several of these ATP-competitive IKKβ antagonists, including PS-1145, were shown to induce intrinsic toxicity [83,84]. As a result of these reported systemic toxicities, there are still no FDA-approved IKKβ antagonists to date. Additionally, due to the continuous focus on developing IKKβ inhibitors, pharmacological tools which can selectively inhibit IKKα activity have been less abundant.

Given the tremendous breakthroughs which have uncovered the role of IKKα in oncogenesis, studies have explored strategies to inhibit IKKα, particularly in prostate cancer. Apigenin, a plant flavone, has been shown to directly bind to IKKα, resulting in decreased IKKα phosphorylation with a lack of effects on IKKβ activity, and decreased p65 expression and activation in PC-3 cells [85]. Additionally, following corchorusoside C treatment, total IKKα and p65 expression was reduced in zebrafish and NIK expression was attenuated in DU-145 prostate cancer cells [86].

In 2017, a series of selective and potent ATP-competitive IKKα small molecule inhibitors was published [87]. In July 2024, the structure and mechanism of action of these first-in-class selective IKKα antagonists were published [88]. The IKKα antagonist in this series with the highest binding affinity for IKKα, SU1261 (IKKα Ki = 10 nM, IKKβ Ki = 680 nM), was shown to successfully inhibit downstream IKKα-dependent signalling events within the non-canonical NF-κB signalling pathway, including p100 phosphorylation [88]. These advances in targeting IKKα within cell-based experiments are undoubtedly intriguing, but, ultimately, the effectiveness of these IKKα antagonists are currently unexplored in *in vivo* models. Moreover, their effect on downstream IKKα-dependent genes and potential drug-related toxicity has yet to be evaluated. Toxicological studies *in vivo* are required to determine whether these novel IKKα inhibitors will induce less adverse effects than previously clinically trialled IKKβ inhibitors. There is a continuous challenge to deliver novel drugs into *in vivo* models and replicate results from cell-based studies, but nevertheless, these compounds offer a new specific method to target IKKα without targeting IKKβ.

## 11. Conclusion

**Figure 3.**
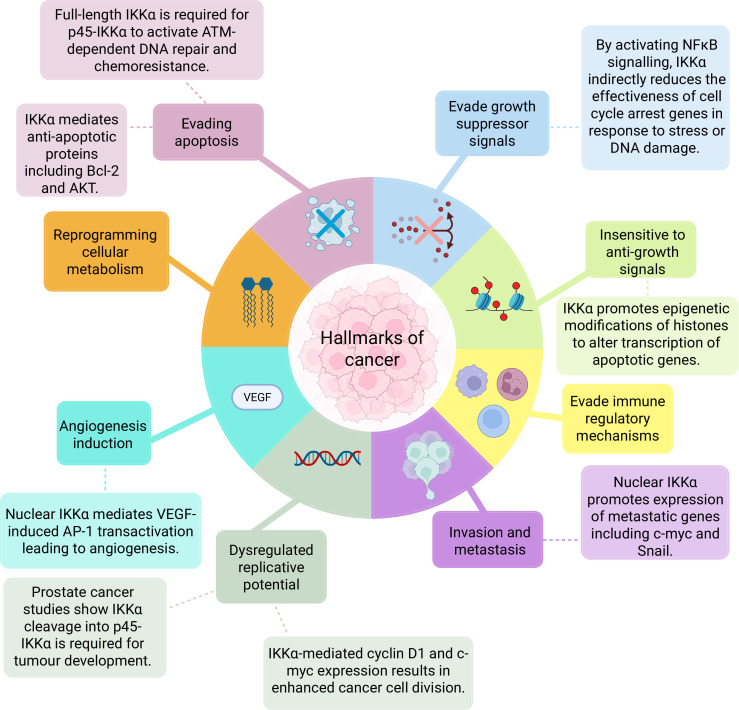
A summary diagram of the roles of IKKα in hallmarks of cancer. This figure depicts a summary of some examples of the mechanisms by which IKKα promotes cancer hallmarks, which in turn promotes tumour progression. Original figure created on BioRender.com.

Overall, a growing body of evidence has demonstrated that the role of IKKα extends beyond its originally characterized function in non-canonical NF-κB signalling. IKKα contributes to promoting several hallmarks of cancer at the molecular level through diverse mechanisms, operating from both the cytoplasm and nucleus, despite its variable subcellular localization and the existence of two isoforms (figure 3). Notably, while p45-IKKα has been studied in the context of CRC, further research is needed to determine whether its function is CRC specific or broadly relevant across other malignancies.

While there was a historical focus on therapeutically targeting IKKβ over IKKα, the emergence of first-in-class, commercially available, selective IKKα inhibitors open a promising avenue for investigation. However, *in vivo* studies carried out in mice models have revealed that IKKα plays essential roles in skin development, B-cell maturation, secondary lymphoid organ development and tissue homeostasis [23,89,90], raising concerns about systemic inhibition. Therefore, rigorous *in vivo* evaluation of these inhibitors is critical to assess both therapeutic potential and possible on-target toxicity. Such studies will be essential in determining whether the benefits of inhibiting IKKα outweigh the potential risks associated with systemic IKKα inhibition, particularly given its direct and indirect roles in driving oncogene expression. Furthermore, *in vitro* work utilizing the IKKα inhibitors to define IKKα-dependent gene networks in cancer-specific cell types would also be beneficial to clarify the overall role of IKKα in oncogene expression. Moreover, *in vivo* testing of these IKKα inhibitors remain a priority, particularly considering the protein’s fundamental involvement in physiological processes. Future strategies aiming to inhibit IKKα in a cell- or tissue-specific manner by utilizing targeted drug delivery systems may enhance therapeutic efficacy while minimizing systemic toxicity, an approach that could outperform previous efforts targeting IKKβ [84].

In essence, IKKα represents *'double trouble*' in cancer by contributing to oncogenesis through cytosolic functions in NF-κB signalling and nuclear roles in directly regulating oncogene expression. Understanding and disentangling these dual roles will be essential for developing safe, effective therapies that leverage the full potential of IKKα inhibition.

## Data Availability

Supplementary material is available online [91].
